# Individual transcriptional activity of estrogen receptors in primary breast cancer and its clinical significance

**DOI:** 10.1002/cam4.41

**Published:** 2012-10-30

**Authors:** Tatsuyuki Gohno, Yuko Seino, Toru Hanamura, Toshifumi Niwa, Mitsuyo Matsumoto, Nobuo Yaegashi, Hanako Oba, Masafumi Kurosumi, Hiroyuki Takei, Yuri Yamaguchi, Shin-ichi Hayashi

**Affiliations:** 1 Department of Molecular and Functional Dynamics, Graduate School of Medicine, Tohoku UniversityAoba-ku, Sendai, 980-8575, Japan; 2 Research Institute for Clinical Oncology, Saitama Cancer CenterIna-machi, Saitama, 362-0806, Japan; 3 Department of Gynecology, Graduate School of Medicine, Tohoku UniversityAoba-ku, Sendai, 980-8575, Japan; 4 Department of Pathology, Saitama Cancer CenterIna-machi, Saitama, 362-0806, Japan; 5 Division of Breast Surgery, Saitama Cancer CenterIna-machi, Saitama, 362-0806, Japan; 6 Center for Regulatory Epigenome and Disease, Graduate School of Medicine, Tohoku UniversityAoba-ku, Sendai, 980-8575, Japan

**Keywords:** Breast cancer, ERE transcriptional activity, estrogen receptor α, Ki67, Luminal A

## Abstract

To predict the efficacy of hormonal therapy at the individual-level, immunohistochemical methods are used to analyze expression of classical molecular biomarkers such as estrogen receptor (ER), progesterone receptor (PgR), and HER2. However, the current diagnostic standard is not perfect for the individualization of diverse cases. Therefore, establishment of more accurate diagnostics is required. Previously, we established a novel method that enables analysis of ER transcriptional activation potential in clinical specimens using an adenovirus estrogen response element–green fluorescence protein (ERE-GFP) assay system. Using this assay, we assessed the ERE transcriptional activity of 62 primary breast cancer samples. In 40% of samples, we observed that ER protein expression was not consistent with ERE activity. Comparison of ERE activity with clinicopathological information revealed that ERE activity was significantly correlated with the ER target gene, PgR, rather than ER in terms of both protein and mRNA expression. Moreover, subgrouping of Luminal A-type breast cancer samples according to ERE activity revealed that ERα mRNA expression correlated with ER target gene mRNA expression in the high-, but not the low-, ERE-activity group. On the other hand, the low-ERE-activity group showed significantly higher mRNA expression of the malignancy biomarker Ki67 in association with disease recurrence in 5% of patients. Thus, these data suggest that ER expression does not always correlate with ER transcriptional activity. Therefore, in addition to ER protein expression, determination of ERE activity as an ER functional marker will be helpful for analysis of a variety of diverse breast cancer cases and the subsequent course of treatment.

## Introduction

To predict the efficacy of hormonal therapy for breast cancer at the level of the individual, immunohistochemical methods are used to analyze classical molecular biomarkers such as estrogen receptor (ER), progesterone receptor (PgR), and HER2 [1–3]. Novel markers such as Ki67, FOXA1, and GATA3 are also examined and used to predict long-term outcome after neoadjuvant endocrine treatment [4–7]. However, the current diagnostic standard is not always suitable for the classification of cases. In ER-positive patients, endocrine therapy to antagonize ER signaling is ineffective in approximately 30% of cases [8]. This discrepancy could be the result of the activation of other ER-independent estrogen-related signaling pathways in these breast cancer cells, such as insulin-like growth factor 1 (IGF-1)- or vascular endothelial growth factor (VEGF)-mediated signaling cascades [9, 10]. Therefore, reliable diagnostic techniques or tools are required for the sensitive evaluation of likely endocrine therapy efficacy for individual patients.

ER is activated by estrogen [11, 12] or protein phosphorylation by kinases such as mitogen-activated protein kinase (MAPK) and phosphatidylinositol 3-kinase (PI3K)/Akt [13, 14]. Activated ER induces transcription of genes containing the estrogen response element (ERE). The molecular mechanisms regulating transcriptional activity by ER have been well investigated in breast cancer cells. However, although ER protein expression has been evaluated by immunohistochemistry (IHC) [1, 2], its relationship with ERE transcriptional activity has not been reported. We have previously observed several cases in which ER protein expression and ER target gene mRNA expression do not correlate [15–18]. These results suggest that ER protein expression may not necessarily reflect the function of ER.

To explore the possibility of recategorizing breast cancers, we analyzed human breast cancer cases according to three features: ER protein expression, ERE transcriptional activity, and ER target gene mRNA expression. We have previously produced a construct in which the common ERE is ligated upstream of green fluorescence protein (GFP) cDNA, and packaged into an adenovirus vector [12, 19, 20]. Primary breast cancer cells, prepared from patients, were infected with this adenovirus vector, and the ERE transcriptional activity was measured by analyzing the GFP fluorescence, as previously described for endometrial cancer [20]. We also determined the protein and mRNA expression levels of ER and the ER target genes identified in our microarray [18, 15–17], using formalin-fixed paraffin-embedded (FFPE) sections from the same patients. This is the first report describing the relationship between ER and its transcriptional activity using clinical samples. Our result indicates that Luminal A-type breast cancer may be classified into two or more types. These findings could be used for a novel predictive model of hormonal therapeutic effectiveness. Indeed, further subtyping of Luminal A-type breast cancer based on the functional evaluation of ER could contribute to more accurate diagnosis and the selection of more effective treatment strategies.

## Materials and Methods

### Tumor samples

Primary human breast cancer tissues were surgically obtained from 62 informed and consenting patients at the Saitama Cancer Center Hospital (Saitama, Japan) between 2005 and 2007 (Table 1) with approval from the Saitama Cancer Center and Tohoku University Ethics Committee (Saitama Cancer Center No. 216, Tohoku University No. 2008-442). These living cells were used for the assessment of ERE activity. FFPE sections were also prepared from these samples and used for hematoxylin and eosin staining, immunohistochemical staining, and real-time reverse transcription polymerase chain reaction (PCR). Preparation of FFPE and staining were carried out as previously [21] described.

**Table 1:** Patient clinicopathological information

Characteristic	*n*
Age	
<50	27
≥50	35
Menopausal	
Pre	28
Post	33
No (men)	1
Tumor size (mm)	
<20	27
≥2	30
Unknown	5
Stage	
0	3
I	13
II	33
III	5
Unknown	8
Grade	
1	7
2	9
3	33
Unknown	13
ER	
Positive	46
Negative	13
Unknown	3
PgR	
Positive	46
Negative	13
Unknown	3
HER2	
Positive	10
Negative	47
Unknown	5

### Reagents

ICI 182,780 (Fulvestrant, pure antiestrogen) and 4-hydroxytamoxifen (Tamoxifen) were purchased from Sigma-Aldrich (St. Louis, MO).

### IHC of the ER, PgR, and HER2

**Figure 1:**
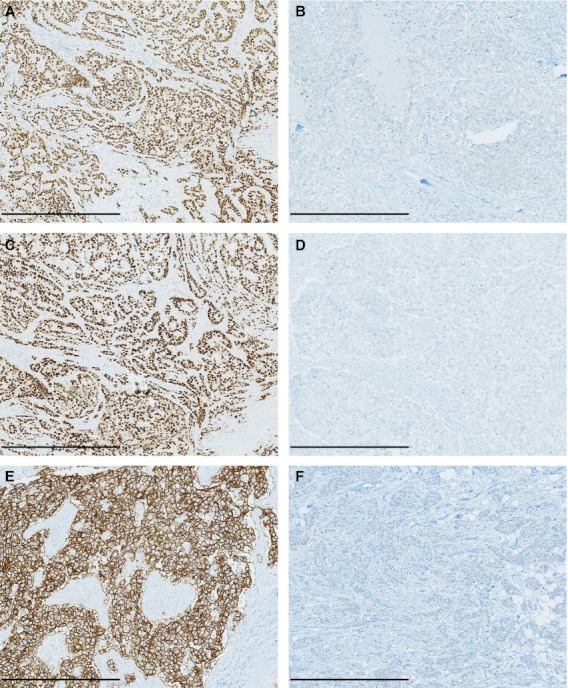
Representative images of IHC labeling of ER (A: positive; B: negative), PgR (C: positive; D: negative), and HER2 (E: positive; F: negative). Scale bars, 500 μm.

We analyzed the expression of ER and PgR by IHC. ER was detected using monoclonal anti-ERα antibody 1D5 (M7047; Dako, Glostrup, Denmark), and PgR using monoclonal antibody PgR 636 (M3569; Dako). Immunointensity was graded on the basis of Allred scoring [22] (ER: Fig. 1A and B; PgR: Fig. 1C and D). We also assessed HER2 positivity using the HercepTest™ (Dako) and scored the results as 0, 1, 2, and 3, according to the ASCO/CAP guidelines [1, 2] (Fig. 1E and F). A HER2-positive status was defined as HER2 protein 3 or 2 and FISH ratio of more than 2.2. Histologic grading was evaluated according to the Elston and Ellis grading scheme [23] with slight modification.

### ERE transcriptional activity assay in primary tumor cells: Ad-ERE-GFP assay

To assess ERE transcriptional activity in primary tumor cells, we used the Ad-ERE-GFP assay [12, 19, 20]. The isolation of tumor cells was performed as previously described by Ackerman [24] with slight modifications. Briefly, cancer tissue specimens were minced to ∼1 mm^3^ in size after being rinsed with phosphate-buffered saline (PBS), and digested with collagenase solution (1 mg/mL collagenase, 40 mg/mL bovine serum albumin, 2 mg/mL glucose, 1× antibiotic-antimycotic, and 50 μg/mL gentamicin in HBSS [Hank's balanced salt solution]) for 20–30 min at 37°C. The cells, including tumor cells, were washed several times with PBS, and incubated in 24-well plates with 400 μL of PRF-RPMI (phenol red-free RPMI) 1640 medium (GIBCO BRL, Grand Island, NY) supplemented with 10% fetal calf serum (Tissue Culture Biologicals, Tulare, CA). The cells were then infected with 2 × 10^9^ PFU (plaque forming unit) (in 293A cells) Ad-ERE-GFP, and incubated for a further 3 days at 37°C in 5% CO_2_–95% air. To examine the infectivity of the adenovirus in primary tumor cells, the cells were infected with 2 × 10^9^ PFU Ad-ERE-GFP or Ad-CMV-DsRed. Approximately 80% of cells were confirmed to be infected. To evaluate drug sensitivity, the cells were simultaneously treated with or without ICI 182,780 or 4-hydroxytamoxifen at a final concentration of 1 μmol/L at the time of infection. To quantify the GFP expression level, the number of cancer cells expressing GFP was counted under a fluorescence microscope after harvesting by treatment with trypsin. The pathologist checked that only cancer cells expressed GFP. All experiments were done in duplicate, and the ERE activity was determined by the percentage of cells expressing GFP.

### Total RNA preparation and real-time reverse transcription PCR

RNA was extracted from 40 μm FFPE sections containing a large tumor site using RecoverAll™ Total Nucleic Acid Isolation (Ambion, Austin, TX) according to the manufacturer's instructions after paraffin removal with xylene. The RNA concentration from FFPE samples was determined using the NanoDrop spectrophotometer (Thermo Scientific, Waltham, MA). Total RNA (0.5 or 1 μg) was converted to first-strand cDNA primed with a random hexamer in a 20 μL reaction volume using a TaKaRa RNA PCR Kit (AMV) Ver.3.0 (TaKaRa Bio Inc., Otsu, Japan). An aliquot of this solution (2 or 4 μL) was used as a template for real-time reverse transcription PCR to quantify the mRNA expression levels of ER and several ER target genes that were identified in our previous study [15–17] (Table 2) using the StepOne™ Real-Time PCR System (Applied Biosystems Inc., Foster City, CA). The PCR thermal settings were as follows: initial denaturation at 95°C for 10 min followed by 40 amplification cycles of 95°C for 15 sec, and annealing and elongation at 60°C for 1 min. The primer sequences used in this study are listed in Table 2.

**Table 2:** Primers used for real-time PCR

Gene	Sequence
RPL13A	F: 5′-CCT GGA GGA GAA GAG GAA AG-3′
	R: 5′-TTG AGG ACC TCT GTG TAT TT-3′
Bcl-2	F: 5′-GTG GAT GAC TGA GTA CCT GAA C-3′
	R: 5′-GCC AGG AGA AAT CAA ACA-3′
Efp	F: 5′-CAT CTC TCA AGG CCA AGG-3′
	R: 5′-GCT ACT GTA TAG CAC TCT GAG A-3′
EGR3	F: 5′-GAG CAG TTT GCT AAA CCA AC-3′
	R: 5′-AGA CCG ATG TCC ATT ACA TT-3′
ERα	F: 5′-CTC CCA CAT CAG GCA CAT-3′
	R: 5′-CTC CAG CAG CAG GTC ATA-3′
HDAC6	F: 5′-GTC TAC TGT GGT CGT TAC ATC-3′
	R: 5′-GGC CTG ACA GTA GTA ACA C-3′
IGFBP4	F: 5′-CCA CGA GGA CCT CTA CAT CAT AC-3′
	R: 5′-ACA CAC CAG CAC TTG CCA C-3′
IGFBP5	F: 5′-TCT CTG CAC CTG AGA TGA GA-3′
	R: 5′-GTC ACA ATT GGG CAG GTA-3′
Ki67	F: 5′-GTC TCT GGT AAT GCA CAC TC-3′
	R: 5′-TCC ACA TGG ATT TCT GAA C-3′
PgR	F: 5′-AGC TCA CAG CGT TTC TAT CA-3′
	R: 5′-CGG GAC TGG ATA AAT GTA TTC-3′

### Statistical analysis

Statistical analysis for comparison of two independent groups was performed with the Mann–Whitney *U* test and the StatFlex 6.0 software program (Artech Co., Ltd., Osaka, Japan). For comparison among three groups or more, the Kruskal–Wallis test was used. Correlation coefficients were also calculated with StatFlex 6.0. Data are expressed as mean ± standard deviation. *P* < 0.05 was considered statistically significant.

## Results

### Human breast cancer clinical samples exhibit varying ERE transcriptional activity and drug sensitivity

**Figure 2:**
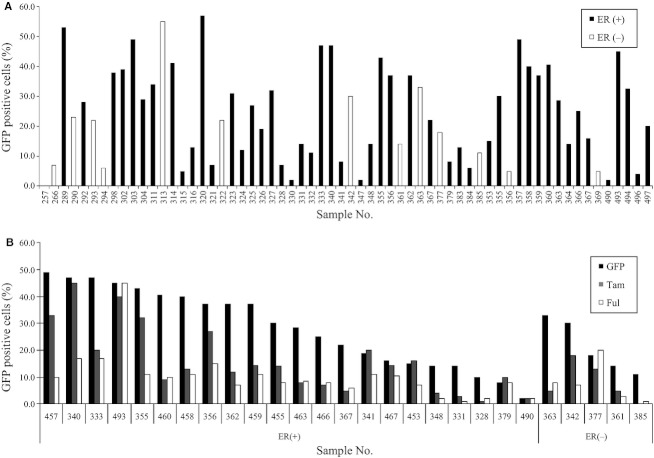
ERE transcriptional activity of primary breast tumor cells. (A) Primary breast tumor cells were infected with Ad-ERE-GFP and incubated for 3 days. Cells expressing GFP were then counted. Black bars represent ER-positive samples and white bars represent ER-negative samples. (B) Ad-ERE-GFP infected cells simultaneously received ethanol (EtOH; black bars), 4-hydroxytamoxifen (Tam; gray bars), and ICI 182,780 (Ful; white bars) at a final concentration of 1 μmol/L to determine drug sensitivity.

We have previously established an adenovirus-mediated ERE-GFP assay, named Ad-ERE-GFP assay, which enables the quantitative evaluation of endogenous ER transcriptional activity in clinical specimens [12, 19, 20]. Using this assay system, we investigated the ERE transcriptional activity of breast cancer cells isolated from surgical specimens. These clinical samples showed various levels of GFP expression representative of ERE activity, which was not associated with the status of ER (Fig. 2A). The range of the GFP positivity measured for all samples was 0–57%, where the average and median were 23.8% and 20%, respectively. In the ER-positive group alone, the range of GFP positivity was 2–57% (0–55%), and the average and median were 26.2% (17.1%) and 28.5% (18%), respectively. In drug sensitivity tests (Fig. 2B), Tamoxifen (Tam) and Fulvestrant (Ful) treatments effectively reduced ERE transcriptional activity to 75% and 85% of ER-positive samples, respectively; however, some samples were insensitive to either one (representative samples 340, 341, and 453, Fig. 2B) or both drugs (representative samples 493, 467, and 379, Fig. 2B). Notably, some ER-negative samples showed high GFP positivity that was reduced by antiestrogen treatment (representative samples 363, 342, 361, and 385, Fig. 2B). Furthermore, local recurrence was reported for two patients: ER-positive 467 and ER-negative 385. While ER-positive 467 showed low drug sensitivity in our test, ER-negative 385 showed high drug sensitivity. These data reiterate that sensitivity to endocrine therapy is not solely dependent on the status of ER. Thus, these results suggest that IHC to determine the ER status combined with Ad-ERE-GFP assay as an auxiliary diagnostic might more accurately predict the sensitivity of breast cancers to hormonal therapy. Furthermore, some patients defined as ER negative may still be candidates for endocrine therapy.

### ERE transcriptional activity significantly correlates with PgR protein expression

**Figure 3:**
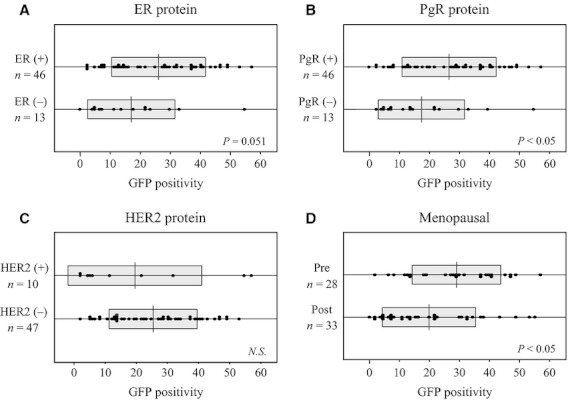
Comparative analysis of GFP positivity in 62 primary breast tumor samples by clinicopathological information. These box plots show the intergroup comparison of (A) ER protein expression, (B) PgR protein expression, (C) HER2 protein expression, and (D) menopausal status.

We next assessed the relationship between ERE transcriptional activity and clinicopathological information including ER, PgR, and HER2 protein expression as assessed by IHC (Fig. 3). ER protein expression appeared to correlate with ERE transcriptional activity, but this was not statistically significant (Fig. 3A). In contrast, ERE transcriptional activity was significantly correlated with the protein expression of PgR, an ER target gene (Fig. 3B). HER2 protein expression, on the other hand, did not correlate with ERE transcriptional activity (Fig. 3C). We also examined whether ERE transcriptional activity might be associated with other clinical information including age and tumor grade and whether patients were pre- or postmenopausal. In this analysis, ERE transcriptional activity was only correlated with postmenopausal status (Fig. 3D); age and tumor grade did not associate with ERE transcriptional activity. The malignant phenotype, however, such as tumor size or higher clinical stage, tended to show low-ERE transcriptional activity (data not shown). The positive correlation of ERE transcriptional activity with PgR protein suggests that our Ad-ERE-GFP assay reliably reflects ERE transcriptional activity and tumor malignancy as ER functional target. Additionally, because Ad-ERE-GFP uses only ERE as readout of ER-driven transcriptional activity, it is more specific than PgR, which is influenced by many transcriptional cofactors.

### ER target gene expression does not correlate with ERE transcriptional activity

**Figure 4:**
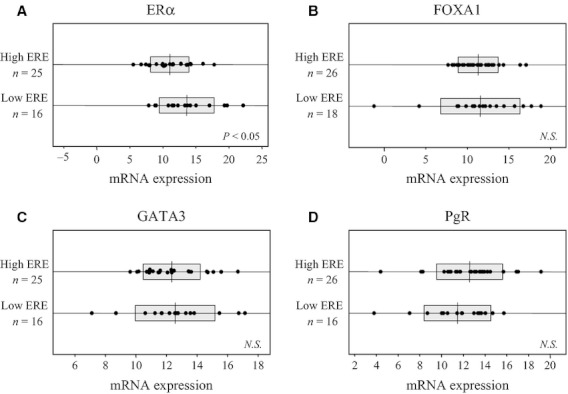
The intergroup difference of ERα and its related or target gene mRNA expression in 46 ER-positive breast tumor samples divided into high- or low-ERE transcriptional activity groups. These box plots show the intergroup differences of (A) ERα; (B and C) ER-related genes: (B) FOXA1, (C) GATA3; and (D) ER target gene: PgR.

Next, we focused on the relationship between ER protein expression and ERE transcriptional activity. According to our previous studies [25, 26], samples with no less than 20% GFP positivity were designated as having high-ERE transcriptional activity. Using this threshold, samples were divided into two groups of high- and low-ERE transcriptional activity. We then compared ERE transcriptional activity, from the high and low groups, with mRNA expression levels of ER and three ER target genes, FOXA1, GATA3, and PgR, in ER-positive cases (Fig. 4). Statistical analysis uncovered significant intergroup differences in ER mRNA expression. ER mRNA expression was significantly higher in the low-ERE group than in the high-ERE group (Fig. 4A). Although PgR mRNA expression was not significantly different between low- and high-ERE groups, there was a tendency for mRNA expression to be higher in the high-ERE-activity group than in the low-ERE-activity group that was in agreement with protein expression analysis (Figs. 3B and 4D). For the other ER target genes examined (Efp, EGR3, HDAC6, IGFBP4, and IGFBP5), mRNA expression levels were not significantly different between low- and high-ERE transcriptional activity groups (data not shown). FOXA1 (Fig. 4B) and GATA3 (Fig. 4C), two genes recently proposed to be related to Luminal-type breast cancer [5–7], also showed no significant difference in mRNA expression regardless of the level of ERE transcriptional activity (FOXA1, *P* = 0.786; GATA3, *P* = 0.689). Therefore, our data suggest that ER target gene expression is not correlated with ERE transcriptional activity. Thus, the regulation of ER target genes is likely not solely dependent on ER, but could instead involve the convergence of other signaling pathways.

### ERE transcriptional activity suggests there are two distinct classes of Luminal A-type breast cancer

**Figure 5:**
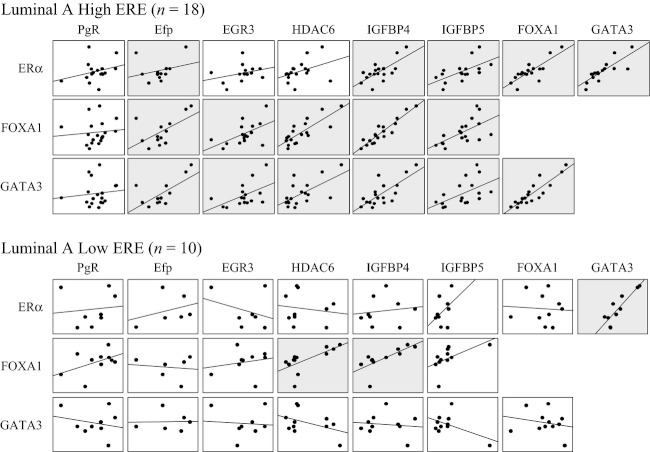
Correlation diagrams of ERα and ER target genes in 28 Luminal A-type breast tumor samples divided into high- or low-ERE transcriptional activity groups. The dots in each square represent the mRNA expression of each gene, and the straight lines show the correlation graphs. The gray squares represent significant correlation (*P* < 0.05), and the white squares reflect no significant correlation.

Because no significant difference in FOXA1 and GATA3 mRNA expression was observed in the ER-positive group, we decided to explore a more specific breast cancer subtype. Therefore, we conducted correlation analysis of ER and its target genes in Luminal A group breast cancer (Fig. 5). Analysis of this subset of ER-positive breast cancer specimens unveiled that ERα mRNA expression levels significantly correlated with Efp, IGFBP4, IGFBP5, FOXA1, and GATA3 in the high-ERE group, but not in the low-ERE-group, with the exception of GATA3. Moreover, FOXA1 and GATA3 mRNA levels correlated not only with ERα but also the other ER target genes: Efp, EGR3, HDAC6, IGFBP4, and IGFBP5, in the high-ERE group alone. On the other hand, some ER target genes, HDAC6, IGFBP4, and IGFBP5, significantly correlated with each other in the low-ERE group (data not shown). This result supports the hypothesis that some ER target genes are activated through signal pathways other than ER. These data also suggest that ERE activity can further distinguish Luminal-type breast cancer into two classes. Although there was large variation in the mRNA expression profiles of ER target genes between tumor cases, the determination of ERE transcriptional activity appears to be worthwhile for distinguishing ER function-dependent and -independent cases among Luminal A-type breast cancer.

### Ki67 is strongly inversely correlated with ERE transcriptional activity

**Figure 6:**
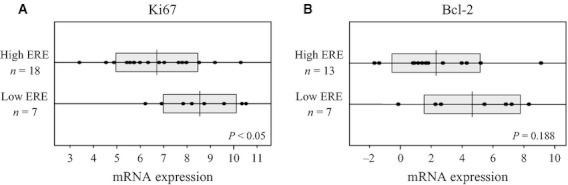
The intergroup difference of Ki67 and Bcl-2 mRNA expression in 28 Luminal A-type breast tumor samples divided into high- or low-ERE transcriptional activity groups. These box plots show the intergroup difference of (A) Ki67 and (B) Bcl-2 mRNA expression levels in each group.

Ki67 [4] and Bcl-2 [27] have been reported to correlate with the malignancy of breast cancer. Therefore, we determined the correlation between ERE transcriptional activity and mRNA expression levels of Ki67 and Bcl-2 in Luminal A breast cancer samples. Interestingly, Ki67 mRNA expression was significantly higher in the low-ERE-activity group than in the high-ERE-activity group (Fig. 6A). Bcl-2 mRNA expression also tended to be higher in the low-ERE-group than in the high-ERE group (Fig. 6B). These genes are recognized as poor prognosis factors, but their mechanisms of action for breast cancer are not well defined. Therefore, further exploration of the relationship between ERE transcriptional activity, Ki67 and Bcl-2 may lead to mechanistic insights and explain why the latter two are higher in the group with low-ERE activity.

## Discussion

ER is one of the most important transcription factors related to malignancy and proliferation in breast cancer. In this study, we focused on the function of ER as a transcription factor and analyzed human-derived breast cancer specimens according to three features: ER protein expression, mRNA expression profiles of ER target genes, and ERE transcriptional activity as an index for ER function. First, we analyzed ERE transcriptional activity in human breast cancer clinical samples by Ad-ERE-GFP assay. Ad-ERE-GFP assay is highly sensitive, even more than luciferase assays. In contrast to FACS, the Ad-ERE-GFP assay requires fewer cells and can measure the ERE activity of living cells in culture. Therefore, this assay is suitable for measuring transcriptional activity of heterogeneous clinical samples. Indeed, using the Ad-ERE-GFP assay, we demonstrated that primary breast cancer tumor cells exhibit various levels of ERE transcriptional activity in spite of ER positivity (Fig. 2A). The GFP fluorescence, an index of ERE transcriptional activity, was reduced by antiestrogen treatment with either Tamoxifen or Fulvestrant in almost all samples (Fig. 2B). However, several samples did not show drug sensitivity, especially to tamoxifen, suggesting that ER antagonism does not always correlate with inhibition of ER target gene transcription. ER genomic effects are activated not only by estrogen but also by its phosphorylation mediated by signaling pathways such as MAPK or PI3K/AKT pathway [12, 13]. The breast cancer cells in which GFP (ERE transcriptional activity) was not reduced in response to antiestrogenic drugs may have adopted these pathways.

Next, we compared ERE transcriptional activity with general clinicopathological information. These analyses revealed that ERE transcriptional activity had a tendency to correlate with ER protein expression levels (Fig. 3A) as well as menopausal status, but these data were not statistically significant. In contrast, a significant correlation was observed between ERE transcriptional activity and PgR protein expression levels (Fig. 3B). PgR protein expression has been clinically used for evaluating the function of ER activity [1], as confirmed by the present result with Ad-ERE-GFP assay. However, ERE transcriptional activity remains a better readout of ER function as PgR is just one many ER target genes and is regulated by many other transcription factors such as Sp1 or AP-1 [28, 29]. Additionally, the Ad-ERE-GFP assay excludes the influence of other transcription factors and therefore more directly reflects the function of the ER protein than PgR. Our results also demonstrated that ERE transcriptional activity does not correlate with ER protein expression. Together with the results of the drug sensitivity tests mentioned above, our data suggest that not only ER protein expression but also its functional evaluation should be determined to more accurately decide the treatment with most likely efficacy for ER-positive breast cancers.

To more fully investigate the relationship of ERE transcriptional activity to ER status and ER target gene expression, we classified ER-positive primary breast cancer samples into two groups of high- and low-ERE transcriptional activity as evaluated by Ad-ERE-GFP assay. Of note, the low-ERE-activity group had significantly higher ER mRNA expression levels than the high-ERE-activity group. In terms of expression levels of the six ER target genes examined, there were no significant intergroup differences between high- and low-ERE-activity groups. These results suggest that there is a group in which ER does not effectively transmit estrogen signaling, in spite of high-ER protein expression. This may be because the ERE transcriptional activity is intercepted downstream, or different feedback mechanisms may exist for each target gene. Therefore, analyzing ERE transcriptional activity may help determine whether and how much the breast cancer depends on ER signaling.

Because many Luminal A-type breast cancers were contained in ER-positive samples, we extracted the Luminal A group from the ER-positive group and investigated its mRNA expression profiles (Fig. 5). FOXA1 and GATA3 have recently been reported to be associated with the Luminal type [5, 6, 26], and ER protein expression level clearly reflected their mRNA expression levels, especially for GATA3. Although the mRNA expression of both genes was not significantly different regardless of ERE transcriptional activity when all ER-protein-positive tumors were examined (Fig. 4B and C), subclassification of Luminal-type breast tumors into low- and high-ERE-activity revealed that these two groups had different correlation tendencies between ERα, FOXA1, and GATA3 mRNA expression levels and ER target genes. These results suggest that ERE activity can classify the Luminal A-type into two distinctions, whereby determination of ERE transcriptional activity may support the assessment of endocrine therapy efficacy. More interestingly, Ki67 and Bcl-2 tended to be higher in the low-ERE-activity group in ER-positive breast cancer (Fig. 6). Ki67 expression is a validated index of malignancy in breast cancer [3]. At the time of this research, local recurrence was found in two patients included in the Luminal A group. Both patients were also from the low-ERE-group, with measured GFP positivity of 7% and 16%, respectively. Although further work is required, the discrepancy in Ki67 and ERE transcriptional activity may help to explain the relationship between Ki67 and breast cancer.

It is widely known that there are individual differences in endocrine therapy efficacy despite ER positivity [2]. In this study, recategorization of breast cancer by ERE transcriptional activity suggests the possibility of distinguishing groups for whom endocrine therapy would be effective and ineffective. The range of treatment choices could also be expanded, especially in Luminal A-type breast cancer patients. We expect that ERE transcriptional activity could become an additional or surrogate marker for analysis of ER protein function and subsequently the improved treatment of breast cancer.
